# Adiponectin improves clozapine-induced lipid accumulation and inflammation without affecting insulin resistance

**DOI:** 10.1515/biol-2025-1333

**Published:** 2026-07-20

**Authors:** I-Lun Tsai, Shih-Chao Lin, Lin Lin, Pei-Shan Tsai, Shiow-Yi Chen

**Affiliations:** Department of Bioscience and Biotechnology, National Taiwan Ocean University, Keelung, 202301, Taiwan; Bachelor Program in Marine Biotechnology, National Taiwan Ocean University, Keelung, 202301, Taiwan; Marine Biotechnology and Environmental Ecology Sustainability Department, National Taiwan Ocean University, Keelung, 202301, Taiwan

**Keywords:** clozapine, adiponectin, lipid accumulation, NF-κB phosphorylation, HepG2 cells

## Abstract

Clozapine, an atypical antipsychotic, is effective for treatment-resistant schizophrenia but frequently causes metabolic adverse effects, including hepatic lipid accumu-lation, inflammation and insulin resistance. Adiponectin, an adipocyte-derived cytokine with anti-inflammatory and insulin-sensitizing properties, may counteract these effects; however, its ability to mitigate clozapine-induced hepatic alterations remains unclear. This study examined whether adiponectin overexpression reduces clozapine-induced lipid accumulation, inflammatory signaling, and whether it restores insulin-related Akt signaling in human HepG2 liver cells. Cells were treated with 25 μM clozapine for 24 or 48 h, and adiponectin was overexpressed by plasmid transfection. Lipid accumulation was quantified by BODIPY staining. AdipoR1 and AdipoR2 expression was analyzed by qPCR, and protein levels of FASN, phosphorylated NF-κB, and phosphorylated Akt were assessed by Western blotting. Clozapine increased lipid accumulation, upregulated FASN, and reduced AdipoR1 and AdipoR2 expression. Adiponectin overexpression significantly decreased lipid levels, which was associated with reduced NF-κB phosphorylation, suggesting attenuation of inflammatory signaling. However, adiponectin did not restore insulin-stimulated Akt phosphorylation. In summary, adiponectin selectively reduced clozapine-induced lipid accumulation and inflammatory signaling in HepG2 cells, whereas impaired insulin-stimulated Akt phosphorylation remained unchanged under the present experimental conditions. These findings indicate a selective protective role of adiponectin and suggest its potential as a modulator of clozapine-induced metabolic side effects.

## 1 Introduction

Schizophrenia is a chronic psychiatric disorder character-ized by impairments in cognition, behavior, and perception, leading to significant functional disability [1]. This condition imposes a considerable burden not only on affected in-dividuals but also on their families emotionally and finan-cially. Pharmacological treatment with atypical antipsychotic drugs (AAPDs) remains the primary approach for disease management. AAPDs are widely prescribed due to their enhanced efficacy and fewer extrapyramidal side effects compared to typical antipsychotics [2].

However, AAPDs are frequently associated with meta-bolic adverse effects, including obesity, insulin resistance, dyslipidemia, impaired glucose tolerance, and type 2 dia-betes, which are collectively recognized as features of metabolic syndrome [3]. Among AAPDs, clozapine is partic-ularly known for inducing severe metabolic disturbances. Patients receiving clozapine have a higher risk of hepatic inflammation and progression to nonalcoholic steatohepa-titis (NASH) or cirrhosis [4].

Despite its clinical significance, the molecular mecha-nisms underlying clozapine-induced metabolic dysregula-tion remain incompletely understood. Previous studies have suggested that antipsychotic drugs, including clozapine, may activate the sterol regulatory element-binding protein (SREBP) pathway, thereby increasing the expression of downstream lipogenic enzymes such as fatty acid synthase (FASN) and acetyl-CoA carboxylase (ACC) [5]. In parallel, clozapine may suppress fatty acid oxidation, further exac-erbating lipid accumulation in hepatic cells [6].

There is increasing evidence that adiponectin, an adi-pokine secreted by adipose tissue, is a critical regulator of hepatic lipid metabolism and insulin sensitivity [7], 8]. Together with other adipokines, such as leptin and interleukin-6 (IL-6), adiponectin plays a key role in main-taining metabolic homeostasis. Mechanistically, adiponectin exerts its metabolic effects primarily through its receptors, AdipoR1 and AdipoR2, which activate downstream pathways such as AMP-activated protein kinase (AMPK) and peroxi-some proliferator-activated receptor α (PPARα) [9], [10], [11]. These pathways regulate fatty acid oxidation, glucose metabolism, and cellular energy homeostasis. In contrast, canonical in-sulin signaling is primarily mediated through the PI3K/Akt pathway. Given that these regulatory processes operate through distinct molecular mechanisms, lipid metabolism and insulin signaling can be differentially regulated or uncoupled under conditions of drug-induced metabolic stress [12]. Clinically, adiponectin has also been proposed as a potential biomarker of metabolic syndrome in patients with schizophrenia treated with clozapine [13], reflecting the marked metabolic dysfunction triggered by this drug. However, the role of adiponectin in clozapine-induced lipid accumulation remains unclear, and direct mechanistic evi-dence linking the underlying mechanisms to clozapine re-mains lacking.

In the present study, we aimed to assess the adverse metabolic impact of clozapine on hepatic cells *in vitro* and to investigate whether adiponectin could mitigate clozapine-induced lipid accumulation and alterations in insulin signaling. By elucidating this relationship, our findings may provide novel insights into the molecular interplay between antipsychotic treatment and hepatic metabolism and sup-port the future development of safer pharmacological interventions.

## 2 Materials and methods

### 2.1 Cell culture and treatments

Human hepatocellular carcinoma HepG2 cells were ob-tained from the Institute of Biochemistry and Molecular Biology, National Yang Ming University, Taiwan. Cells were cultured in Dulbecco’s modified Eagle’s medium (DMEM) supplemented with 10 % fetal bovine serum (FBS), 1 % penicillin-streptomycin, 2 mM l-glutamine, and non-essential amino acids. Cells were maintained at 37 °C in a humidified incubator with 5 % CO_2_. HepG2 cells were seeded at a density of 2 × 10^5^ cells per well in 6-well plates. Cells used in this study were maintained at passages below 10. All cultures were routinely tested for mycoplasma contamina-tion, and only mycoplasma-free cells were used in the experiments.

Clozapine (Sigma-Aldrich, Cat. #C6305, ≥98 % purity) was dissolved in DMSO to a stock concentration of 10 mM and diluted in culture medium to the indicated concentra-tions before use. Recombinant human insulin and other chemicals were purchased from Sigma-Aldrich unless otherwise noted. The final DMSO concentration in the cul-ture medium was maintained below 0.25 % (v/v), and an equivalent volume of DMSO was applied as the vehicle control in all the corresponding experiments.

### 2.2 Cell viability assay

Cell viability of HepG2 cells was assessed using the 3-(4,5-dimethylthiazol-2-yl)-2,5-diphenyltetrazolium bromide (MTT) assay. Cells were seeded in 96-well plates and treated with different concentrations of clozapine for 24 and 48 h. After treatment, MTT solution (final concentration 0.5 mg/mL) was added to each well and incubated for 4 h at 37 °C. The resulting formazan crystals were dissolved in DMSO, and the absor-bance was measured at 570 nm using a microplate reader. Cell viability was expressed as a percentage relative to the vehicle control group.

### 2.3 Plasmid transfection

The human adiponectin cDNA was cloned into a CMV promoter-driven expression plasmid. Transfections were performed using TurboFect™ (Thermo Fisher Scientific, USA) according to the manufacturer’s instructions. To opti-mize transfection conditions in HepG2 cells, preliminary experiments were conducted using a GFP-expressing plasmid to evaluate various DNA-to-reagent ratios. The ra-tio that yielded the highest transfection efficiency with minimal cytotoxicity was selected for subsequent experi-ments. For transfection, 1 µg of DNA and 1 µL of transfection reagent were mixed and added to cells in complete medium containing 10 % fetal bovine serum (FBS). After 6 h of transfection, the medium was replaced, and cells were incubated for an additional 18–24 h prior to subsequent as-says. Adiponectin overexpression was confirmed by West-ern blot analysis (Supplementary Figure 1).

### 2.4 Lipid accumulation assay

HepG2 cells were treated with 25 μM clozapine for 24 or 48 h and then stained with BODIPY 493/503 (Cat. # D3922, Thermo Fisher Scientific) at a final concentration of 1 μg/mL for 30 min at 37 °C. Cells were washed twice with PBS and analyzed by flow cytometry (BD FACSCanto II). Representa-tive images were acquired using fluorescence microscopy (Leica DMi8) and the gating strategy is shown in Supple-mentary Figure 2.

### 2.5 Gene expression analysis

Total RNA was extracted from approximately 2 × 10^5^ HepG2 cells in a 6-well plate using TRIzol reagent (Invitrogen, USA). Total RNA was treated with RNase-free DNase I for 15 min at room temperature, followed by quantifying with NanoDrop spectrophotometer. Subsequently, 1 μg of total RNA was reverse- transcribed in 20 μL reaction using Moloney murine leukemia virus (M-MLV) reverse transcriptase. The resulting cDNA was used for quantitative real-time PCR (qPCR), with a fixed volume of cDNA applied to each reaction. Prior to analysis, a titration assay was performed to confirm that the cDNA input was within the optimal detection range. Gene expression of adiponectin receptors, AdipoR1 and AdipoR2, was analyzed using Roche SYBR Green reagent on a Bio-Rad iQ5 real-time PCR detection system. IL-6 and IL-10 mRNA levels were also analyzed by RT-qPCR using beta-actin as the internal control. Relative expression was calculated by the ΔΔCt method using β-actin as the reference gene. The thermal cycling program consisted of 95 °C for 3 min, then 40 cycles of 95 °C for 10 s and 55 °C for 30 s. The primer sequences are listed in the Supplementary Materials and Methods [14].

### 2.6 Insulin stimulation assay

HepG2 cells were transfected with adiponectin expression plasmid or a control vector and treated with or without 25 μM clozapine for 48 h. Following treatment, cells were serum-starved for 6 h to minimize basal signaling and then stimulated with 100 nM recombinant human insulin (Sigma-Aldrich, Cat. #I2643) for 20 min. Protein lysates were then harvested for Western blotting to assess Akt phosphorylation.

### 2.7 Western blot analysis

Cells were lysed using M-PER^®^ reagent supplemented with protease and phosphatase inhibitors. Equal amounts of protein were separated by SDS-PAGE and transferred onto PVDF membranes. Membranes were blocked with 5 % non-fat milk and incubated overnight at 4 °C with primary antibodies (1:1,000 dilution) against LC3B (#2775), FASN (#3180), p–NF–κB p65 (Ser536, #3033), NF-κB p65 (#8242), p-Akt (Ser473, #4060), p-ACC (Ser79, #3661), Akt (pan) (#4691) and β-actin (#4970). Membranes were then incubated with HRP-conjugated secondary antibodies (1:2,000 dilution, #7074). All antibodies were purchased from Cell Signaling Technology. Signals were detected using ECL substrate and X-ray film exposure. Band intensities were quantified with ImageJ software (NIH) and normalized to β-actin as a loading control.

### 2.8 Statistical analysis

All experiments were performed in at least three indepen-dent biological replicates. Data are presented as mean ± standard deviation (SD). Statistical analyses were performed using GraphPad Prism v9.0 software. For com-parisons among three or more groups, a one-way analysis of variance (ANOVA) was performed, followed by Tukey’s post-hoc test for multiple comparisons. A *p*-value of <0.05 was considered statistically significant.

## 3 Results

### 3.1 Clozapine induced lipid accumulation in HepG2 cells

**Figure 1:**
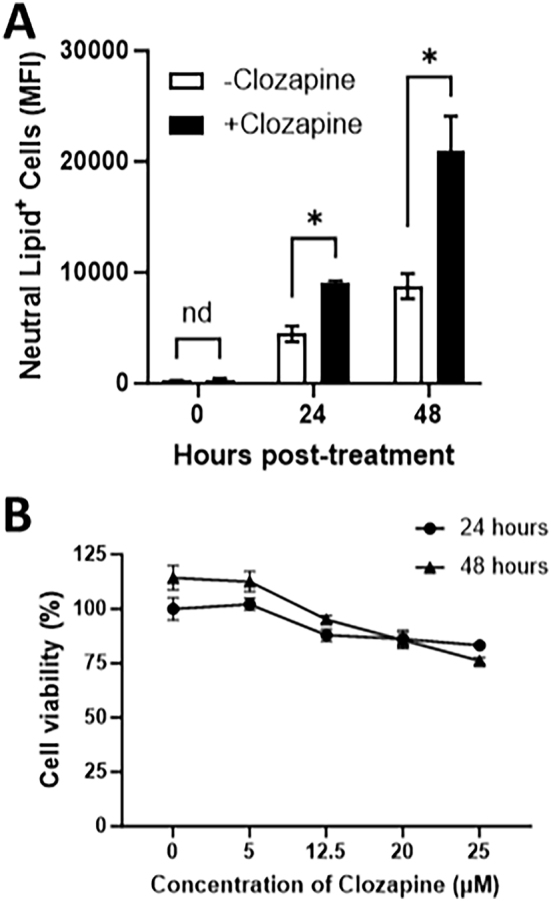
Clozapine induced lipid accumulation in HepG2 cells. HepG2 cells were treated with 25 μM clozapine for 24 or 48 h, followed by staining with BODIPY™ 493/503. Intracellular neutral lipid levels were quantified by flow cytometry and expressed as mean fluorescence intensity (MFI). For each sample, 10,000 events were analyzed. Data are presented as mean ± SD (*n* = 3). *p < 0.05 compared with control.

To investigate whether clozapine induces lipid accumulation in hepatic cells, HepG2 cells were treated with 25 μM cloza-pine for 24 and 48 h. Neutral lipids were then stained with BODIPY 493/503, and fluorescence intensity was quantified by flow cytometry [15]. As shown in Figure 1A, clozapine treatment significantly increased intracellular lipid levels in HepG2 cells in a time-dependent manner, as indicated by the elevated mean fluorescence intensity (MFI). Moreover, MTT assays were performed to evaluate potential cytotoxicity induced by clozapine. Approximately 80 % of the cells remained viable under treatment with the highest clozapine concentration at 24 or 48 h (Figure 1B), suggesting that the observed lipid accumulation was unlikely to be primarily caused by cytotoxicity.

**Figure 2:**
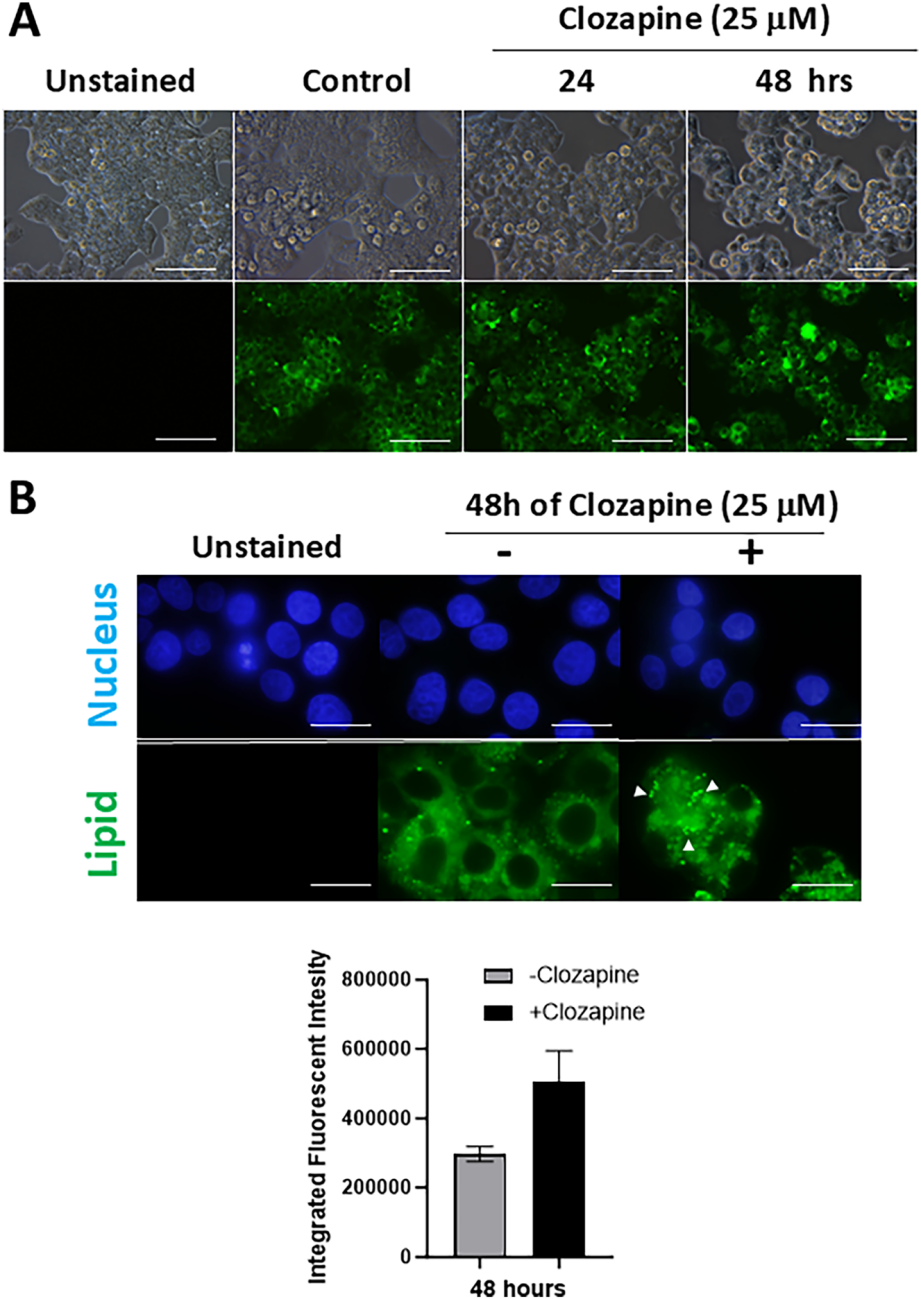
Clozapine increases intracellular lipid droplet accumulation in HepG2 cells. HepG2 cells were treated with clozapine at the indicated concentrations for 24 and/or 48 h, followed by staining with BODIPY™ 493/503. (A) Fluorescence microscopy images show BODIPY-positive HepG2 cells with or without clozapine treatment. Scale bar: 100 µm. (B) Intracellular localization of neutral lipid droplets (green, indicated by white arrows) and nuclei (blue, stained with DAPI) following treatment with 25 μM clozapine for 48 h. The bar graph represents the quantitative analysis of relative fluorescence intensity measured by ImageJ. Scale bar: 25 µm.

To further validate clozapine-induced lipid accu-mulation, the distribution of lipid droplets was examined by fluorescence microscopy. As shown in Figure 2A, clozapine-treated cells displayed a marked increase in lipid droplet signals, both in number and intensity, compared to untreated controls. Consistently, BODIPY-positive lipid droplets were predominantly localized in the cytoplasm and were clearly distinct from DAPI-stained nuclei. Moreover, quantitative analysis showed that lipid droplet abundance was markedly elevated in clozapine-treated cells (Figure 2B), further confirming enhanced intracellular lipid accumulation.

### 3.2 Clozapine altered lipid metabolism-related signaling in HepG2 cells

**Figure 3:**
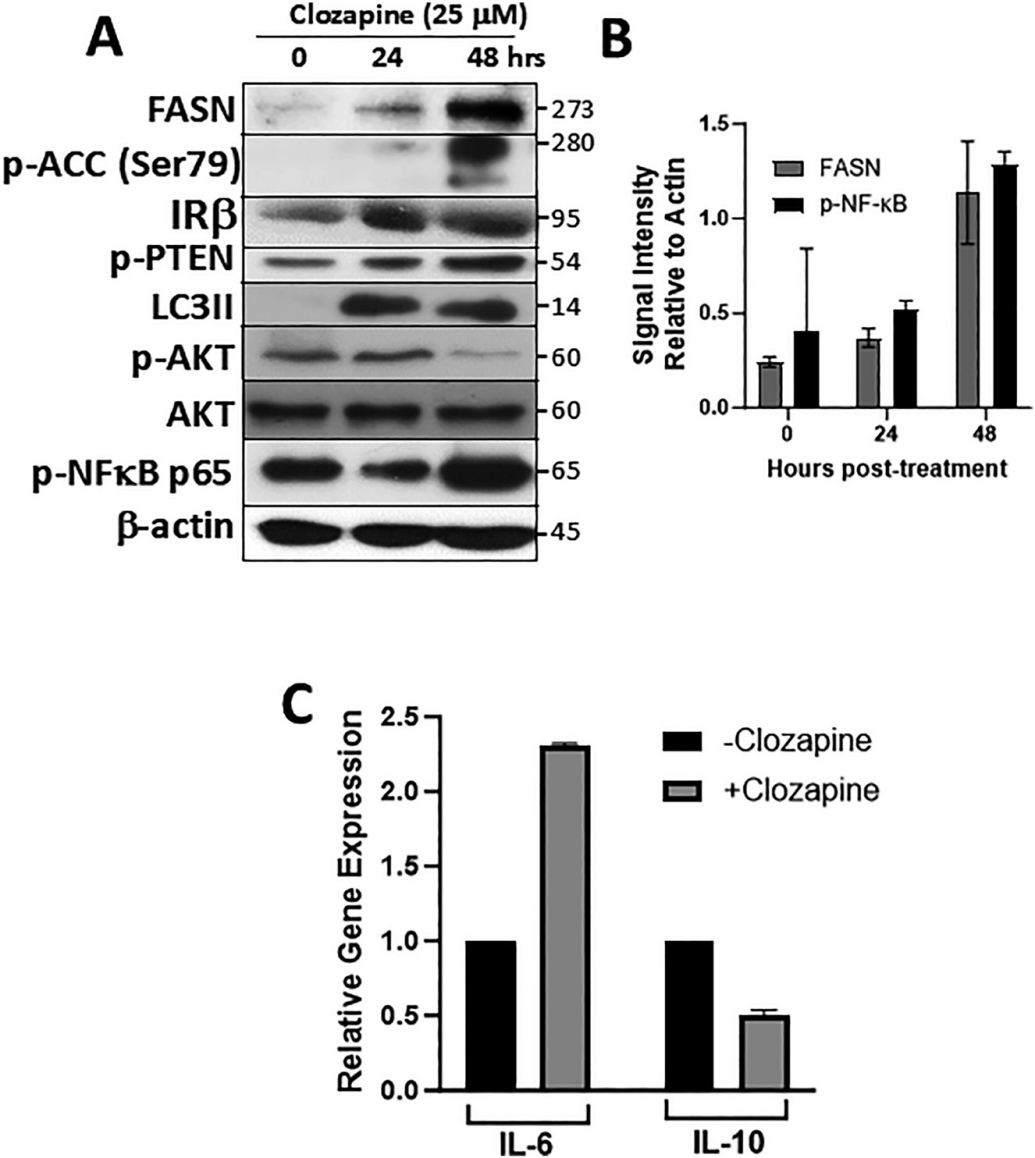
Clozapine alters lipid metabolism-, inflammatory-, and insulin signaling–related proteins in HepG2 cells. (A) HepG2 cells were treated with 25 µM clozapine for 24 or 48 h, and proteins involved in fatty acid synthesis, insulin signaling, and inflammatory responses were analyzed by western blotting. (B) Quantification of fatty acid synthase (FASN) and phosphorylated NF-κB at indicated time points. Protein levels were normalized to β-actin as a loading control and expressed relative to the untreated control. (C) Relative mRNA expression levels of IL-6 and IL-10 in HepG2 cells treated with or without 25 μM clozapine for 48 h, as determined by RT-qPCR. Transcript levels were normalized to β-actin as an internal control. Data are presented as the mean ± SD from three independent experiments (*n* = 3). Statistical significance was determined by one-way ANOVA followed by Tukey’s post hoc test. *p < 0.05.

To further examine the molecular changes associated with clozapine-induced lipid accumulation, we analyzed the expression of lipid metabolism- and stress-related proteins in HepG2 cells treated with or without clozapine for 24 and 48 h by Western blotting. As shown in Figure 3A, clozapine treatment increased the expression of fatty acid synthase (FASN), phosphorylated acetyl-CoA carboxylase (p-ACC), and insulin receptor-β (IRβ). The increase in FASN suggests enhanced fatty acid synthesis and is consistent with the observed lipid accumulation. However, because p-ACC generally represents the inactive form of the ACC [16], its elevation might reflect a compensatory metabolic response to clozapine-induced lipid stress. The concurrent increase in IRβ further suggests that clozapine may alter insulin-related metabolic signaling in HepG2 cells.

Moreover, clozapine treatment upregulated phosphor-ylated PTEN and LC3-II, which are associated with stress re-sponses, including altered PI3K/Akt signaling and autophagosome accumulation. In parallel, a reduction in phosphorylated Akt (p-Akt) expression was observed, suggesting impaired insulin signaling (Figure 3A). Notably, phosphorylated NF-κB (p-NF-κB), a central regulator of inflammation, was also elevated in a time-dependent manner during clozapine treatment (Figure 3A and B). Consistently, RT-qPCR showed that clozapine increased IL-6 mRNA expression and decreased IL-10 mRNA expression in HepG2 cells (Figure 3C). Together, these data suggest that clozapine not only promotes intracellular lipid accumulation but also induces inflammatory and stress responses in HepG2 cells.

### 3.3 Clozapine altered adiponectin receptor expression in HepG2 cells

**Figure 4:**
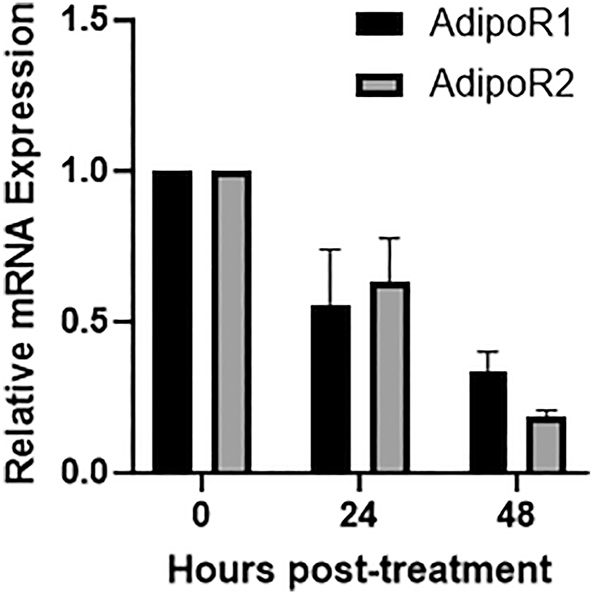
Clozapine reduces adiponectin receptor expression in HepG2 cells. HepG2 cells were treated with 25 μM clozapine for 24 or 48 h. The mRNA expression levels of adiponectin receptors AdipoR1 and AdipoR2 were measured by quantitative RT-PCR. Gene expression levels were normalized to β-actin and expressed relative to the untreated control. Data are presented as the mean ± SD.

Adiponectin is a well-established adipokine involved in the regulation of lipid metabolism and insulin sensitivity [17]. To determine whether clozapine interferes with adiponectin signaling, we measured the gene expression levels of adiponectin receptors, AdipoR1 and AdipoR2, in HepG2 cells following clozapine treatments. Quantitative PCR analysis showed that clozapine significantly suppressed both AdipoR1 and AdipoR2 mRNA levels in a time-dependent manner, with a more pronounced reduction observed at 48 h after treatment (Figure 4). These data suggest that clozapine downregulates the gene expression of adiponectin receptors, which may contribute to impaired adiponectin-related metabolic regulation in HepG2 cells.

### 3.4 Adiponectin attenuated clozapine-induced lipid accumulation in HepG2 cells

**Figure 5:**
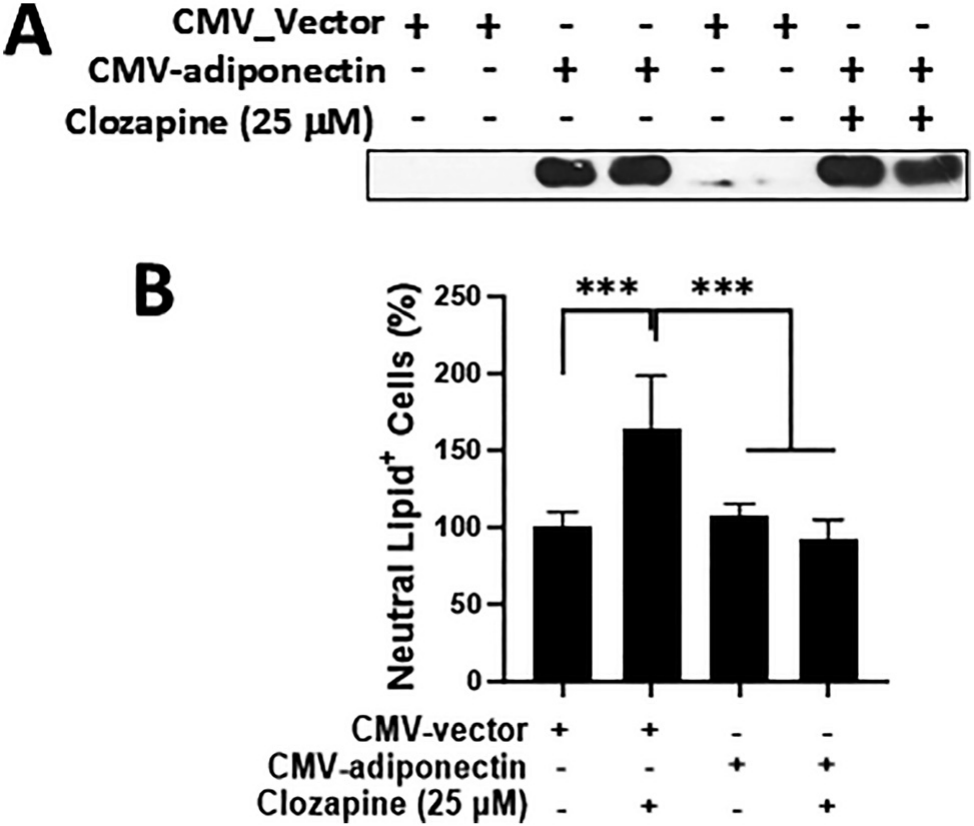
Adiponectin attenuates clozapine-induced lipid accumulation in HepG2 cells. (A) The protein expression of adiponectin was confirmed by western blotting in the presence and absence of clozapine at 48 h of treatment. (B) HepG2 cells were transfected with either the adiponectin expression plasmid or control vector and treated with or without clozapine for 48 h. The neutral lipid-positive cells were stained with BODIPY™ 493/503 and analyzed by flow cytometry. Data are presented as the mean ± SD. *** *p* < 0.001.

To further elucidate the mechanistic role of adiponectin in clozapine-treated hepatocytes, HepG2 cells were transfected with an adiponectin expression plasmid driven by the cytomegalovirus (CMV) promoter to overexpress adiponectin. Western blot analysis confirmed successful adiponectin overexpression compared to CMV vector controls. In addition, clozapine treatment did not significantly alter adiponectin protein expression in adiponectin-overexpressing cells (Figure 5A).

Following the confirmation, we assessed the lipid accu-mulation in HepG2 cells with or without adiponectin overexpression and/or clozapine treatment for 48 h by flow cytometry. As shown in Figure 5B, clozapine-treated HepG2 cells without adiponectin overexpression showed a significantly increased percentage of neutral lipid-positive cells. In contrast, adiponectin overexpression attenuated this effect, reducing the percentage of neutral lipid-positive cells to a level comparable to that observed in cells without clozapine treatment. Importantly, adiponectin overexpression alone did not significantly alter lipid levels in untreated cells, indicating its regulatory effect was more evident under clozapine-induced lipid stress (Figure 5B). Together, these data strongly support a protective role for adiponectin in modulating clozapine-induced lipid dysregulation in HepG2 cells.

### 3.5 Adiponectin attenuated clozapine-induced NF-κB-associated inflammation but did not improve insulin resistance in HepG2 cells

**Figure 6:**
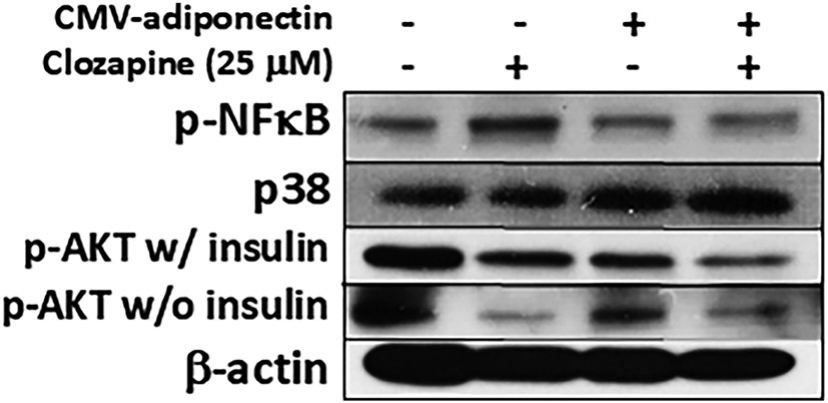
Adiponectin attenuates clozapine-induced NF-κB-associated inflammatory signaling but does not restore insulin-stimulated Akt phosphorylation in HepG2 cells. HepG2 cells were transfected with or without adiponectin and treated with or without 25 μM clozapine for 48 h. Following treatment, cells were serum-starved for 6 h and then stimulated with 100 nM insulin for 20 min prior to protein extraction. NF-κB p65 phosphorylation and insulin-stimulated Akt phosphorylation were analyzed by western blotting.

Considering that obesity is regarded as a chronic inflam-matory state [12] and that adiponectin is known for its anti-inflammatory effects [13], we investigated whether adiponectin could attenuate clozapine-induced inflammatory signaling. Western blot analysis indicated that clozapine markedly increased the phosphorylation of NF-κB, but not p38 MAPK (Figure 6). Notably, adiponectin overexpression reduced clozapine-induced p-NF-κB levels, suggesting that adiponectin attenuated NF-κB-associated inflammatory signaling in clozapine-treated HepG2 cells.

In contrast, adiponectin overexpression did not restore Akt phosphorylation in clozapine-treated cells, even after insulin stimulation (Figure 6). This finding suggests that adiponectin alone was insufficient to rescue clozapine- impaired insulin-related Akt signaling under the current experimental conditions. Together, these observations indicate that adiponectin may mitigate clozapine-induced lipid accumulation and NF-κB-associated inflammatory signaling, but does not restore impaired insulin-stimulated Akt activation in HepG2 cells.

## 4 Discussion

Clozapine is a highly effective antipsychotic for treatment-resistant schizophrenia but is frequently linked to serious metabolic side effects, including hepatic lipid accumulation and insulin resistance [18]. In this study, we demonstrated that clozapine induced significant lipid accumulation and inflammatory signaling in HepG2 cells, accompanied by increased FASN expression, increased ACC phosphorylation, and reduced Akt phosphorylation. These findings align with previous studies reporting that clozapine promoted lipogenesis and disrupted metabolic homeostasis [4], 19].

Our data show that clozapine-treated HepG2 cells exhibited increased FASN expression and intracellular lipid accumulation, supporting enhanced lipogenic responses in HepG2 cells. The elevated level of ACC phosphorylation at Ser79 observed in Figure 3A is consistent with previous reports in clozapine-associated metabolic studies [20]. Notably, because ACC phosphorylation is generally associated with the inhibition of its enzymatic activity, the increase in p-ACC likely reflects compensatory or stress-related metabolic regulation in response to lipid accumulation, rather than direct activation of ACC-mediated fatty acid synthesis. Similar regulatory patterns have been reported in patients with schizophrenia receiving olanzapine, in whom lipid accumulation or hyperphagia occurs concomitantly with feedback inhibition of ACC and dysregulated AMPK signaling [21], 22]. However, given that total ACC levels were not assessed in the present study, the interpretations of changes in ACC phosphorylation should be made with caution.

The concurrent increase in IRβ expression and decrease in Akt phosphorylation, observed alongside stable total Akt levels, suggests a dissociation between insulin receptor expression and downstream Akt activation. The stable expression of total Akt suggests that the reduction in phosphorylated Akt was unlikely to result from a loss of the total Akt protein pool, but rather from impaired activation of the Akt phosphorylation cascade. Clozapine-induced inflammatory response, as reflected by elevated p-NF-κB, together with increased PTEN phosphorylation, may contribute to reduced signal transduction from the insulin receptor to Akt (Figure 3). In this context, the elevation of IRβ does not necessarily reflect enhanced signaling; instead, it could serve as another compensatory cellular response attempting to overcome the downstream blockade of the Akt activation in our model.

In addition to increased FASN expression and ACC phosphorylation, clozapine-treated HepG2 cells exhibited decreased phosphorylated Akt levels, accompanied by increased NF-κB phosphorylation and LC3-II expression. The increase in FASN expression is consistent with enhanced lipogenic responses, whereas the elevation of ACC phosphorylation may reflect compensatory or stress-related metabolic regulation rather than direct activation of ACC-mediated fatty acid synthesis. Meanwhile, increases in NF-κB phosphorylation and LC3-II expression suggest activation of inflammatory and cellular stress responses, including autophagosome accumulation, in hepatic cells (Figure 3) [23], [24], [25], [26]. Furthermore, clozapine significantly reduced the mRNA expression levels of the adiponectin receptors AdipoR1 and AdipoR2 (Figure 4), suggesting that clozapine may impair adiponectin-related metabolic regulation at the receptor-expression level. Overexpression of adiponectin in HepG2 cells selectively attenuated clozapine-induced lipid accumulation and NF-κB-associated inflammatory signaling (Figure 5), supporting a protective role for adiponectin against clozapine-induced metabolic and inflammatory disturbances.

Previous studies have shown that activation of adiponectin receptors suppressed lipid accumulation and prevented obesity following the introduction of additional adiponectin genes into mice [27]. Moreover, clozapine has been shown to reduce the expression of adiponectin and its receptors in 3T3-L1 adipocytes [19]. In line with these reports, our findings demonstrate that clozapine significantly suppressed the mRNA expression of both AdipoR1 and AdipoR2 receptors at 24 and 48 h after treatment (Figure 4), indicating that clozapine may impair adiponectin-related metabolic regulation not only in adipocytes but also in liver cells. Because adiponectin receptors play essential roles in regulating fatty acid oxidation and glucose metabolism, their downregulation may further exacerbate the risk of metabolic disorders such as obesity and type 2 diabetes mellitus [28], 29]. However, it is important to note that although our data demonstrate a significant clozapine-induced downregulation of AdipoR1 and AdipoR2 at the transcriptional level, we did not assess their corresponding protein expression or surface localization. Because mRNA levels do not always perfectly correlate with functional protein abundance, these transcriptional findings should be interpreted with caution.

Although adiponectin overexpression successfully attenuated clozapine-induced lipid accumulation (Figure 5B), it did not concomitantly restore Akt phosphorylation. This divergence suggests that adiponectin may alleviate lipid accumulation through mechanisms that are at least partly independent of the PI3K/Akt pathway. Adiponectin is known to activate metabolic pathways such as AMPK and PPARα, which are involved in fatty acid oxidation and lipid homeostasis. Therefore, the reduction in lipid accumulation observed in adiponectin-overexpressing cells may be mediated through these alternative metabolic regulatory pathways rather than through restoration of Akt phosphorylation. The persistent suppression of p-Akt, despite elevated adiponectin, suggests that clozapine may impair insulin-related Akt signaling through mechanisms that are not fully rescued by adiponectin under the present experimental conditions. Given that obesity is increasingly recognized as a chronic low-grade inflammatory state [30], we speculated that adiponectin overexpression may counteract clozapine-induced inflammatory stress. Indeed, our results showed that adiponectin-overexpressing HepG2 cells exhibited reduced NF-κB phosphorylation following clozapine treatment (Figure 6), suggesting attenuation of NF-κB-associated inflammatory signaling. Together, these findings suggest that adiponectin may exert protective effects by modulating clozapine-induced lipid accumulation and NF-κB-associated inflammatory signaling, consistent with its reported metabolic and anti-inflammatory functions [31], 32].

It should be noted that there are several limitations to the present study. First, while our data demonstrate significant clozapine-induced downregulation of AdipoR1 and AdipoR2 at the transcriptional level, we did not assess their corresponding protein expression or surface localization. Because mRNA levels do not always correlate perfectly with functional protein abundance, these findings should be interpreted with caution. Second, the use of an adiponectin overexpression model in HepG2 cells may generate supraphysiological levels of this adipokine in the culture system. However, we did not explicitly quantify the levels of secreted adiponectin in the culture medium, and the observed cellular responses might have been amplified compared to endogenous states. Furthermore, functional downstream readouts of the adiponectin signaling axis, such as AMPK phosphorylation or PPARα target gene expression, were not evaluated in the present study. Although Akt phosphorylation was examined as a marker of insulin-related signaling, additional upstream and downstream components of the insulin signaling pathway were not comprehensively assessed. Future studies using *in vivo* approaches or more physiological models, including treatment with recombinant human adiponectin, will be necessary to comprehensively elucidate the impact of clozapine on this metabolic axis.

In summary, our study identifies adiponectin as a selective modulator of clozapine-induced lipid accumulation and NF-κB-associated inflammatory signaling in HepG2 cells, while highlighting its inability to restore impaired insulin-stimulated Akt activation under the present experimental conditions. These findings underscore the complexity of clozapine-induced metabolic dysfunction and suggest that adiponectin-based therapeutic strategies may have limitations when used alone in patients receiving clozapine treatment. Nevertheless, our study may help guide the development of future pharmacological interventions aimed at minimizing the metabolic side effects of clozapine and ultimately improving long-term outcomes in psychiatric care.

## 5 Conclusions

Our study demonstrates that clozapine induces lipid accumulation, NF-κB-associated inflammatory signaling, and impaired insulin-stimulated Akt activation in HepG2 cells. Adiponectin overexpression reduces clozapine-induced lipid accumulation and NF-κB phosphorylation, suggesting a partial protective effect against clozapine-induced metabolic and inflammatory stress. However, adiponectin overexpression does not restore insulin-stimulated Akt phosphorylation, as Akt phosphorylation remains suppressed. These results suggest that adiponectin may provide partial protection against clozapine-induced metabolic stress, especially lipid-related effects, but additional strategies may be needed to address insulin resistance.

## Supplementary Material

Supplementary Material Details

Supplementary Material Details

Supplementary Material Details

Supplementary Material Details
