# Supplementary figures and images for: Adiponectin improves clozapine-induced lipid accumulation and inflammation without affecting insulin resistance
Source: Open Life Sci. 2026 Jul 20;21(1):20251333. doi: 10.1515/biol-2025-1333 (PMC13377577; doi:10.1515/biol-2025-1333)

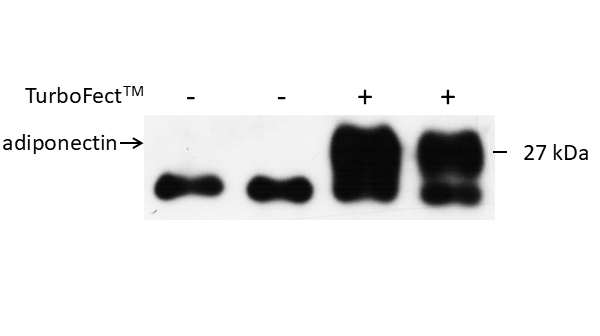

Supplement: Supplementary file 1 — Supplementary Material Details [file j_biol-2025-1333_suppl_001.zip › j_biol-2025-1333_suppl_001.tif]

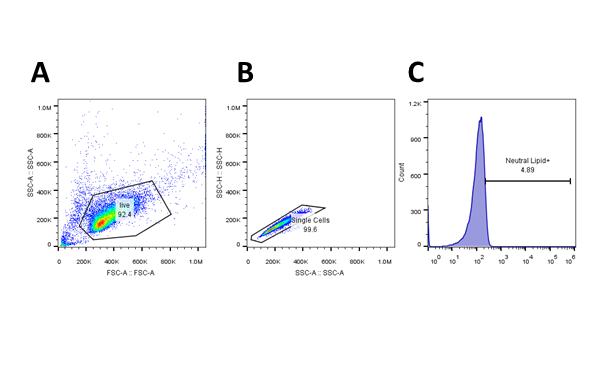

Supplement: Supplementary file 2 — Supplementary Material Details [file j_biol-2025-1333_suppl_002.zip › j_biol-2025-1333_suppl_002.tif]
