# Supplementary material for: Adiponectin improves clozapine-induced lipid accumulation and inflammation without affecting insulin resistance
Source: Open Life Sci. 2026 Jul 20;21(1):20251333. doi: 10.1515/biol-2025-1333 (PMC13377577; doi:10.1515/biol-2025-1333)
Supplement: Supplementary file 3 — Supplementary Material Details [file j_biol-2025-1333_suppl_003.docx]

**Supplementary Materials and Methods**

**Primers for quantitative PCR**

Custom DNA primers for AdipoR1, AdipoR2, and β-actin were synthesized by StarMoon Gene Co., Ltd. (order PO-PR-100603D9; June 3, 2010) and purified by OPC purification. Primer specificity was confirmed using Primer-BLAST, which returned a single hit for each assay. Melt curves analysis showed a single peak, and agarose gel confirmed a single band of the expected size. The assays target AdipoR1 (NM_001290553.2) and AdipoR2 (NM_024551.5), with amplicons sizes of 80 and 87 bp, respectively. No amplification was observed in the no-reverse-transcription (RT) controls for all primer sets. Each 20 µL qPCR reaction contained 200 nM primers, 1–10 ng cDNA and Roche SYBR Supermix. No amplification was detected in the no-template control (NTC; Cq > 40; no melt peak)

Primers:

AdipoR1:

sense, 5 ′-AACGACTATCTGCTACAT-3′;
antisense, 5′-GTATGAATGCGGAAGATG-3′.
AdipoR2:

sense, 5′-GCATCTGTTCTATCTTCC-3′;
antisense, 5′-CATAAAGCCCTCATCTTC-3′.
β-actin (access no. BC113036.1):
sense, 5′-CTGTGCTATCCCTGTACGCC-3′;
antisense, 5 ′-CACGGTTCGTCGTCTCGG-3′.
