# Supplementary material for: Adiponectin improves clozapine-induced lipid accumulation and inflammation without affecting insulin resistance
Source: Open Life Sci. 2026 Jul 20;21(1):20251333. doi: 10.1515/biol-2025-1333 (PMC13377577; doi:10.1515/biol-2025-1333)
Supplement: Supplementary file 4 — Supplementary Material Details [file j_biol-2025-1333_suppl_004.docx]

**Supplementary Figure 1.** **Validation of adiponectin protein expression following plasmid transfection.** HepG2 cells were transfected with either a control vector (-) or a human adiponectin-expressing plasmid (+) for 24 h. Exogenous adiponectin protein expression was confirmed by Western blot analysis using a specific anti-adiponectin antibody, with a prominent band detected at approximately 27 kDa in the transfected groups.

**Supplementary Figure 2.** **Flow cytometry gating strategy for the quantification of intracellular lipid accumulation. (A)** Representative flow cytometry dot plot illustrating the initial identification of intact cells. Cellular debris and dead cells were excluded based on forward scatter area (FSC-A) and side scatter area (SSC-A). **(B)** Cell populations from panel (A) were further analyzed using side scatter-height (SSC-H) versus side scatter-area (SSC-A) to identify singlets **(C)** Representative histogram of BODIPY 493/503 fluorescence intensity within the gated single-cell population. The shift in mean fluorescence intensity (MFI) reflects the relative changes in lipid accumulation between treatment groups.
